# Structure-Based Regulatory Role for the 5′UTR of RCNMV RNA2

**DOI:** 10.3390/v15030722

**Published:** 2023-03-10

**Authors:** Jennifer S. H. Im, Jasmine R. Sheppard, K. Andrew White

**Affiliations:** Department of Biology, York University, Toronto, ON M3J 1P3, Canada

**Keywords:** plant virus, RNA virus, RNA structure, translation, replication, red clover necrotic mosaic virus, dianthovirus, tombusviridae

## Abstract

Red clover necrotic mosaic virus (RCNMV) is a segmented positive-strand RNA virus consisting of RNA1 and RNA2. Previous studies demonstrated that efficient translation of RCNMV RNA2 requires de novo synthesis of RNA2 during infections, suggesting that RNA2 replication is required for its translation. We explored a potential mechanism underlying the regulation of replication-associated translation of RNA2 by examining RNA elements in its 5′ untranslated region (5′UTR). Structural analysis of the 5′UTR suggested that it can form two mutually exclusive configurations: a more thermodynamically stable conformation, termed the 5′-basal stem structure (5′BS), in which 5′-terminal sequences are base paired, and an alternative conformation, where the 5′-end segment is single stranded. Functional mutational analysis of the 5′UTR structure indicated that (i) 43S ribosomal subunits enter at the very 5′-end of RNA2; (ii) the alternative conformation, containing unpaired 5′-terminal nucleotides, mediates efficient translation; (iii) the 5′BS conformation, with a paired 5′-end segment, supresses translation; and (iv) the 5′BS conformation confers stability to RNA2 from 5′-to-3′ exoribonuclease Xrn1. Based on our results, we suggest that during infections, newly synthesized RNA2s transiently adopt the alternative conformation to allow for efficient translation, then refold into the 5′BS conformation, which supresses translation and promotes efficient RNA2 replication. The potential advantages of this proposed 5′UTR-based regulatory mechanism for coordinating RNA2 translation and replication are discussed.

## 1. Introduction

The genomes of positive-strand RNA viruses serve as direct templates for both ribosomes that translate viral proteins and viral RNA-dependent RNA polymerases (RdRps) that synthesize minus-strand RNA intermediates, the first step in genome replication. These two processes cannot occur simultaneously on the same RNA template due to their opposing directions, i.e., ribosomes travel in the 5′-to-3′ direction, while viral RdRps travel in the 3′-to-5′ direction. Consequently, regulation of these incompatible processes is required to allow for efficient viral proliferation during infections [1,2,3,4].

**Figure 1:**
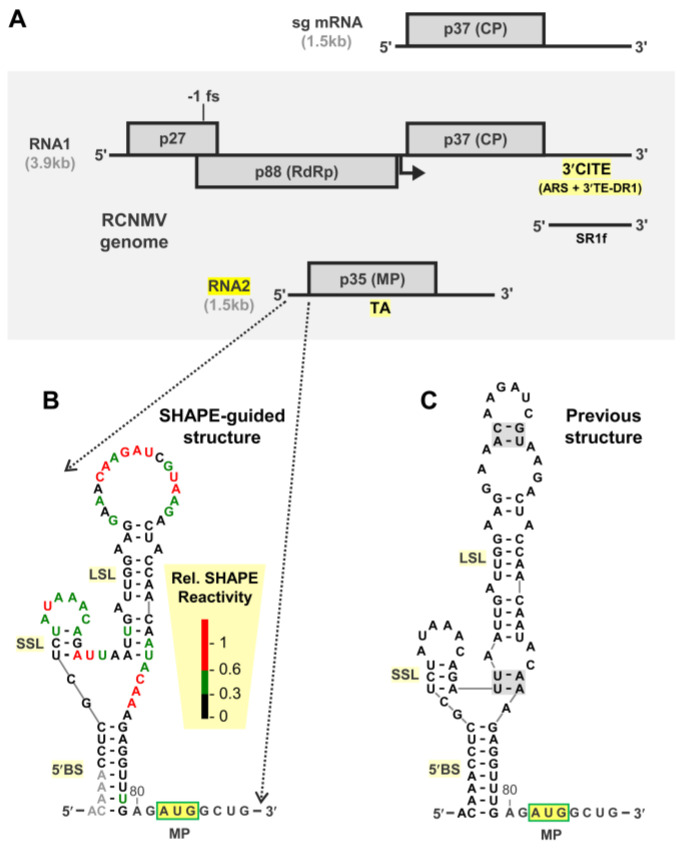
RCNMV genome and 5′UTR structure for RNA2. (**A**) RCNMV genome organization. Structures of RCNMV RNA1 and RNA2 genome segments are shown in the shaded box. Nucleotide lengths are provided in kilobases (kb), and encoded proteins are depicted as rectangles with their corresponding sizes indicated in kilodaltons. The positions of the 3′ cap-independent translation element (3′CITE), the adenine-rich sequence (ARS), and the 3′ translation element of dianthovirus RNA1 (3′TE-DR1) in RNA1 are shown. The location of the trans-activator (TA) in RNA2 is indicated. The transcription initiation site for the sg mRNA in RNA1 is indicated by the angled arrow with the sg mRNA shown above. (**B**) SHAPE-guided model for the 5′UTR of RCNMV RNA2. The 5′-basal stem structure (5′BS), small stem loop (SSL), and large stem loop (LSL) are labeled. Reactivity of color-coded nucleotides in the structure correspond to the relative levels depicted in the key. Grey bases denote null reactivity values. (**C**) Secondary RNA structural model of the 5′UTR of RCNMV RNA2 generated through computational energy minimization [27].

Red clover necrotic mosaic virus (RCNMV; genus *Dianthovirus*, family *Tombusviridae*) possesses a segmented positive-strand RNA genome composed of RNA1 (3.9 kb) and RNA2 (1.5 kb) (Figure 1A) [5,6]. RNA1 encodes three essential open reading frames: an accessory replication protein (p27) [7], the viral RdRp (p88) [8] that is translated through programmed −1 frameshifting [9], and the coat protein (CP), expressed via transcription of a subgenomic (sg) mRNA (1.5 kb) (Figure 1A) [10]. A small noncoding RNA, termed SR1f (0.43 kb) that corresponds a 3′-terminal non-coding segment of RNA1, accumulates during infections and is important for viral accumulation and symptom development (Figure 1A) [11,12]. SR1f corresponds to a stable degradation product of RNA1 (or the sg mRNA) and is generated when a 5′-to-3′ cellular exoribonuclease (e.g., Xrn) stalls at an internal exoribonuclease-resistant RNA structure [11,13]. The second genomic segment, RNA2, is monocistronic and encodes a movement protein (MP) that is required for cell-to-cell and systemic infection in plants [14,15]. The two RCNMV genomic segments also harbor several different RNA elements involved in controlling various aspects of the virus infectious cycle, such as genome replication [16,17,18,19], translation of viral proteins [20,21,22,23], and sg mRNA transcription [10,19,24].

Both RNA1 and RNA2 genomes lack a 5′-cap structure and a 3′-poly(A) tail and thus rely on alternative translation strategies for expression of their viral proteins [6]. Translation of RNA1 is facilitated by two RNA elements in its 3′ untranslated region (3′UTR) that form a 3′ cap-independent translation enhancer (3′CITE) composed of an RNA structure (3′TE-DR1) and an adenine-rich sequence (ARS) that recruit eIF4F and poly(A)-binding protein, respectively (Figure 1A) [20,25,26]. In contrast, translation of RNA2 is mediated by eIFiso4F, and the message does not contain a 3′CITE in its 3′UTR [21,26]. Previous studies on RNA2 determined that its translation is linked to RNA2 replication, implying that only RNA2 genome segments generated de novo during infections can serve as efficient templates for translation [21]. However, exactly how this replication-dependent translation occurs and is regulated remains a mystery.

The 81 nt long 5′UTR of RNA2 was previously investigated and shown to be involved in both RNA2 replication and translation of MP [27]. In that study, complementary nucleotides were identified in the 5′ and 3′ regions of the 5′UTR that could form a helix, which they termed the 5′-basal stem structure (5′BS) (Figure 1B,C) [27]. Some of the results from analyses of the 5′UTR were consistent with formation of 5′BS promoting replication of RNA2 and supressing translation of MP, which implicated the 5′UTR as a regulator of these two processes [27]. Here we have further examined the role of the 5′BS and 5′UTR in modulating replication of RNA2 and translation of its encoded MP. Our results provide compelling evidence supporting a critical role for the 5′BS in modulating these processes and suggest a potential mechanism linking RNA2 replication to activation of translation though conformational changes in its 5′UTR.

## 2. Materials and Methods

### 2.1. Viral Constructs

The full-length cDNA clones of wild-type RCNMV RNA1 and RNA2 genome segments (kindly provided by Tim Sit and Steven Lommel) were used to generate all RCNMV constructs used in this study. R2HA is an RNA2 variant encoding a C-terminally HA-tagged MP. Mutants were derived by standard site-directed PCR-based mutagenesis, using the Q5 High-Fidelity DNA Polymerase kit (NEB). Sequencing was performed to ensure that only the intended modifications were present in mutants. Specific changes introduced into the RCNMV genomes are shown in the accompanying figures.

### 2.2. Preparation of Infectious Viral RNAs

Uncapped viral RNAs were in-vitro-transcribed from SmaI-linearized RCNMV RNA1 and RNA2 constructs using the T7-FlashScribe transcription kit (Cellscript) as described previously [28]. Briefly, 1 ug of DNA template was combined with transcription reagents and incubated at 37 °C for 30 min and then treated with DNase I to digest remaining template DNA. RNA was recovered following precipitation with 5M NH_4_OAc on ice. RNA concentrations were measured by spectrophotometry, and transcript integrity was verified by agarose gel electrophoresis. 5′-monophosphorylated RNAs were generated as described previously [29]. RNA concentrations were measured by spectrophotometry, and transcript integrity was verified by agarose gel electrophoresis.

### 2.3. SHAPE RNA Secondary Structure Analysis

Selective 2′-hydroxyl acylation analyzed by primer extension (SHAPE) [30] was performed on the 5′UTR of full-length RCNMV RNA2 as described previously [2]. Briefly, in vitro transcripts of full-length RNA2 were refolded and then treated with 1-methyl-7-nitroisatoic anhydride. The reaction products then were reverse transcribed with Superscript IV reverse transcriptase (Invitrogen) using fluorescently labeled primers complementary to RNA2 (nt 196–225) and the products separated by capillary electrophoresis. Raw fluorescence intensity data were analyzed using ShapeFinder [31], and nucleotide reactivities were normalized against the average of the top ten peak intensities. SHAPE reactions were performed twice and averaged nucleotide reactivities were used. The RNAStructure web server was employed to integrate reactivities with thermodynamic prediction to generate secondary structure models [32]. RNA secondary structures were created using RNA2Drawer software (version 6.3) [33].

### 2.4. Protoplast Transfections and Viral RNA and Protein Analysis

Cucumber protoplasts were isolated from 6-day-old cotyledons and isolated as described previously [28]. Approximately 3 × 10^5^ protoplasts were transfected with infectious viral transcripts (3 μg of RNA1 and/or 1 μg of RNA2) using PEG-CaCl_2_ [28]. Transfected protoplasts were incubated under constant light at 17 °C for 22 h. Total nucleic acids were purified by extraction with phenol-chloroform isoamyl alcohol (PCI), followed by ethanol precipitation. One-sixth of the total volume was separated in 2% agarose gels and products were transferred to nylon membrane. Plus-strand viral RNA accumulation was detected via Northern blotting using ^32^P-radiolabeled DNA oligonucleotide probes that are complementary to the 3′-end of RNA2 (nt 1222–1242, and 1321–1342), as described previously [19]. Viral RNA accumulation was measured by radioanalytical scanning using a Typhoon FLA 9500 variable mode imager (GE Healthcare) and quantified by Quantity One Software (Bio-Rad).

Viral protein accumulation was detected via Western blotting. Total proteins were separated by 12% sodium dodecyl sulfate polyacrylamide gel electrophoresis (SDS-PAGE) and were transferred to PVDF membrane (Amersham Hybond P 0.45 PVDF). Ponceau S staining was performed to ensure equal loading of total proteins. C-terminally HA-tagged MP was detected with horseradish peroxidase (HRP)-conjugated anti-HA high affinity (3F10) rat monoclonal antibodies (Roche) at 1:4000 dilution. MP-HA bands were detected using ECL Select Western blotting detection reagent (GE Healthcare) and captured through MicroChemi imager (DNR Bio-Imaging Systems). MP levels were calculated relative to that of the reference genome (R1HA) and protein levels were normalized relative to corresponding RNA levels determined through Northern blotting. MP levels were quantified using Quantity One software. Experiments were repeated three times to generate averages and corresponding standard error of the means (SEMs).

### 2.5. In Vitro Translation Assay

Uncapped RNA2 transcripts (0.5 pmol) were assessed for translation using a wheat germ extract in vitro translation system (Promega). MP accumulation was monitored by incorporation of ^35^S-methionine, as described previously [2,34]. Translation products were separated by 12% SDS-PAGE, detected by radioanalytical scanning using a Typhoon FLA 9500 variable mode imager (GE Healthcare), and quantified using Quantity One software (Bio-Rad). MP levels were measured relative to that of R2HA, set as 100%. Translation assays were repeated three times to generate averages and corresponding SEMs.

### 2.6. In Vitro Xrn1 Digestion Assay

In vitro degradation assays were performed as described previously [35] using two micrograms of 5′-monophosphorylated in vitro transcribed RNA2 and purified recombinant yeast Xrn1 (New England Biolabs). Reactions were incubated at 37 °C for 2 h. Following PCI extraction and ethanol precipitation in the presence of glycogen, one-quarter of the recovered RNAs were separated in 2% agarose gels and stained with ethidium bromide. Bands were detected using a Typhoon FLA 9500 variable mode imager (GE Healthcare) and quantified using Quantity One software (Bio-Rad). Each experiment was repeated three times to generate averages and corresponding SEMs.

## 3. Results

### 3.1. Structural Analysis of the 5′UTR of RNA2

The 5′UTR was shown previously to be important for regulating translation of MP from RCNMV RNA2 [27]. In that report, they used a secondary structure model for the 5′UTR that was based solely on computational free energy minimization [36]. We sought to verify or revise this model by adding solution structure probing data for the 5′UTR of RNA2 into the model prediction. SHAPE on the 5′UTR was performed on the full-length RNA2 genome to identify residues with high (i.e., flexible and likely single-stranded) or low (i.e., rigid and likely double-stranded) reactivity [30]. Modification data in the 5′UTR region were then integrated with free energy minimization prediction to generate a more refined RNA secondary structure model [32] (Figure 1B). The SHAPE-guided model predicted the previously defined 5′BS [27] that was formed by base-pairing between the sequences near the 5′- (nts 2–9) and 3′-regions (nts 72–79) of the 5′UTR (Figure 1B). Both models also included an internally positioned small stem loop (SSL) (Figure 1B,C). However, the two models differed in their predictions of an internally positioned large stem loop (LSL). In the previous model, LSL contained additional base pairs in its upper and lower regions (Figure 1C, shaded nucleotides), whereas these residues were predicted to be single-stranded in the SHAPE-guided model (Figure 1B). Thus, except for the differences related to LSL, the two 5′UTR structures were largely consistent.

### 3.2. RNA2 Translation Initiation Occurs through 5′-end Entry and Scanning

**Figure 2:**
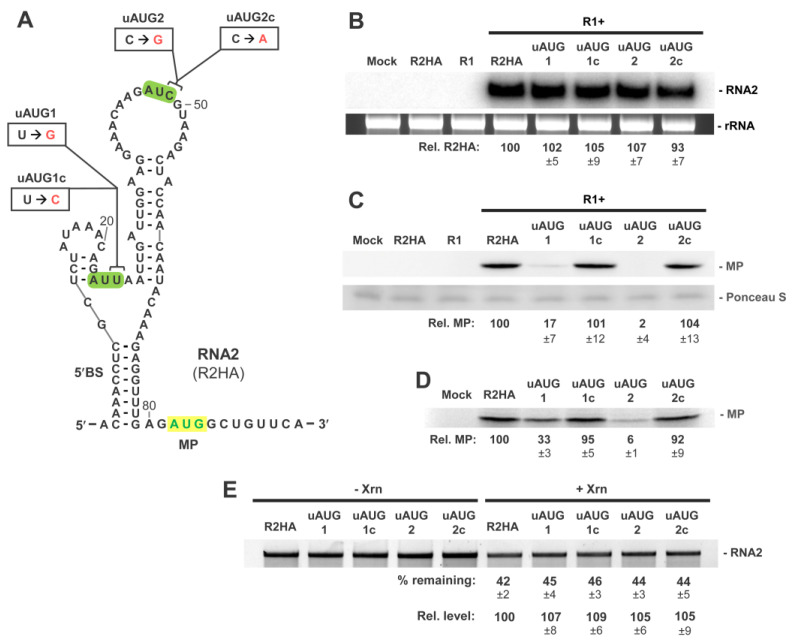
RNA2 upstream AUG (uAUG) mutants assessing 5′-end access. (**A**) RNA secondary structure of the 5′UTR of RNA2 with positions of single nucleotide substitutions that introduce out of frame upstream AUGs (uAUG1, uAUG2) or control non-AUGs (uAUG1c, uAUG2c). Nucleotide substitutions are shown in red and the positions of the uAUGs are highlighted in green. The AUG for the MP ORF is indicated in green nucleotides highlighted in yellow. (**B**) Northern blot analysis of protoplast infections of wt RNA1 (R1) and RNA2 mutants. The position of RNA2 is denoted on the right-hand side along with rRNA loading controls. Relative levels of genome accumulation compared to R2HA (set at 100) are shown with SEMs (n = 3). (**C**) Corresponding Western blot analysis of protoplast infections depicted in panel (**B**). The position of the MP is denoted on the right-hand side along with Ponceau S staining loading controls. Relative levels of MP accumulation normalized to corresponding RNA levels, with that for R2HA set at 100, are shown with SEMs (n = 3). (**D**) In vitro translation in wheat germ lysate of R2HA and uAUG mutants. Relative levels of MP accumulation compared to R2HA (set at 100) are shown with SEMs (n = 3). (**E**) In vitro Xrn1 treatment of R2HA and uAUG mutants. Percentages of input RNA remaining (% remaining) and relative levels (Rel. levels) of RNA remaining compared to R2HA (set at 100) are shown with SEMs (n = 3).

Previous analysis of the 5′UTR of RNA2 indicated that formation of 5′BS inhibited translation of MP, possibly by impeding ribosome entry at the 5′-end [27]. To determine if ribosomes actually access RNA2 via its 5′-terminus, upstream AUGs (uAUGs) were introduced (out of frame, i.e., +1 with respect to the MP ORF) into the 5′UTR at two different locations (Figure 2A). In these modified viral messages, ribosomes entering at the very 5′-end and scanning inward would recognize and initiate at the uAUGs, resulting in decreased translation of MP from its authentic downstream AUG.

The uAUGs and corresponding non-uAUG controls were introduced into the 5′UTR of R2HA (an RNA2 variant encoding a C-terminally HA-tagged MP) by single-nucleotide substitutions, so as to limit effects on higher order structure. Transfection of cucumber protoplasts with wt RNA1 (denoted as R1) and R2HA led to readily detectable accumulation of R2HA RNA in infections via Northern blotting (Figure 2B). Importantly, R2HA and its mutant variants accumulated to similar levels, indicating that the modifications in the latter group did not notably affect their stability or ability to replicate (Figure 2B). Corresponding Western blot analysis of the infections revealed about a fivefold reduction in MP for uAUG1 (~17%) and near zero levels of MP accumulation for uAUG2 (~2%) (Figure 2C). In contrast, control mutations yielded MP levels similar to that for R2HA. Mutant viral genomes were also assessed in vitro with wheat germ extract translation assays, which revealed MP levels that were consistent with those observed in infections (Figure 2D). These translational results indicate that 43S ribosome subunits access RNA2 at or near the very 5′-terminus of the message.

To assess if 5′-end accessibility was altered in the uAUG mutants, the RNA2 transcripts were subjected to the yeast 5′-to-3′ exoribonuclease Xrn1, which requires a minimum of three single-stranded 5′-terminal nucleotides to engage its RNA substrate [37,38]. The results indicated similar levels of Xrn1 resistance in mutant genomes relative to that for R2HA, suggesting comparable degrees of 5′-end accessibility and minimal effect of the single nucleotide substitutions on 5′-terminal RNA secondary structure (Figure 2E). Collectively, these findings indicate that ribosomes enter and begin to scan from the 5′-end of the 5′UTR. Accordingly, formation of the 5′-proximal 5′BS could potentially physically restrict the ability of 43S ribosome subunits to engage RNA2 for productive translation.

### 3.3. The 5′BS Is Required for RNA2 Replication and Inhibits Translation of MP

**Figure 3:**
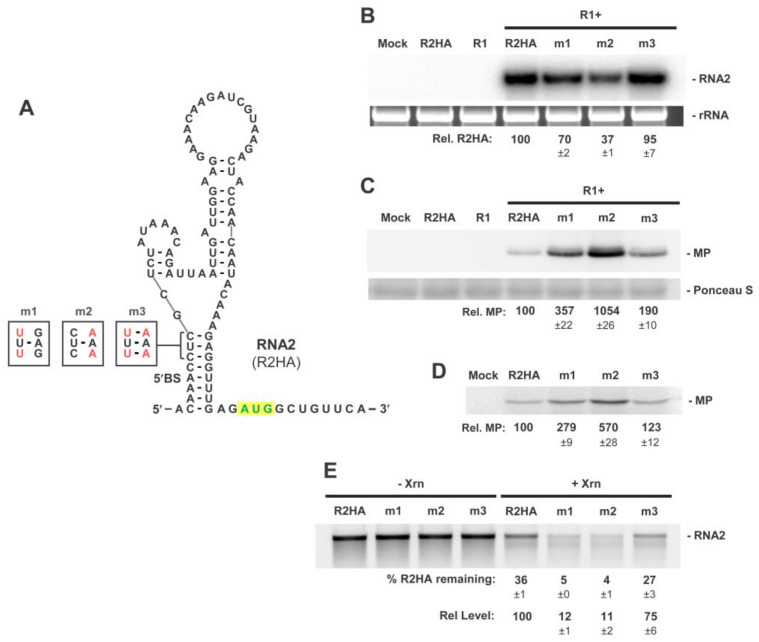
RNA2 5′UTR mutants testing 5′BS function. (**A**) RNA secondary structure of the 5′UTR of RNA2 showing positions of double-nucleotide substitutions designed to inhibit or promote base pairing in the 5′BS. Nucleotide substitutions are shown in red. (**B**) Northern blot analysis of protoplast infections with wt RNA1 (R1) and RNA2 mutants. The position of RNA2 is denoted on the right-hand side along with rRNA loading controls. Relative levels of genome accumulation compared to R2HA (set at 100) are shown with SEMs (n = 3). (**C**) Corresponding Western blot analysis of protoplast infections depicted in panel (**B**). The position of the MP is denoted on the right-hand side along with Ponceau S staining loading controls. Relative levels of MP accumulation normalized to corresponding RNA levels, with that for R2HA set at 100, are shown with SEMs (n = 3). (**D**) In vitro translation in wheat germ lysate of R2HA and mutants. Relative levels of MP accumulation compared to R2HA (set at 100) are shown with SEMs (n = 3). (**E**) In vitro Xrn1 treatment of R2HA and mutants. Percentages of input RNA remaining (% remaining) and relative levels (Rel. levels) of RNA remaining compared to R2HA (set at 100) are shown with SEMs (n = 3).

Based on the finding that ribosomes enter and scan from the 5′-terminus of RNA2 (Figure 2), the possible role of 5′BS in modulating their 5′-access was investigated. Prior analysis showed that preventing 5′BS formation, by deleting or substituting the 3′-half of its stem, enhanced translation, consistent with 5′BS formation being inhibitory [27]. However, it was not clearly established that the base-pairing feature of 5′BS was indeed responsible for the translational repression. Thus, to evaluate the functional importance of secondary structure, compensatory mutational analysis was performed by disrupting (m1, m2) and restoring (m3) base pairing in 5′BS (Figure 3A). Northern blot analysis of protoplasts co-transfected with wt RNA1 and R2HA or one of the compensatory mutants revealed reduced genome accumulation in m1 and m2 (~70%, ~37%, respectively), while compensatory substitutions in m3 restored RNA2 accumulation to near R2HA levels (~95%) (Figure 3B). 5′BS formation therefore favored the accumulation of RNA2. Corresponding Western blot analysis of infections revealed that, conversely, m1 and m2 with destabilized 5′BSs enhanced relative MP accumulation (~357%, ~1054%, respectively), and the compensatory mutation reduced MP translation closer to, but above, R2HA levels (~190%) (Figure 3C). These data confirm the necessity for 5′BS pairing for translational repression. Moreover, the relative levels of MP observed correlated inversely with the strength of the two base pairs targeted for substitution: CGs in wt, 100%; UAs in m3, ~190%; UGs in m1, ~357%; and CAs in m2, ~1054% (Figure 3C). Mutant R2HA viral genomes were also assessed by in vitro translation assays, which revealed a corresponding trend in translational activities (Figure 3D). Thus, both in vitro and in vivo results are consistent with the concept that destabilizing 5′BS allows for greater ribosome access. To further explore 5′-end accessibility in these viral RNAs, Xrn1 assays were performed, and the results indicated that destabilization of 5′BS in m1 and m2 allowed for greater access of Xrn1 to their 5′-ends, as revealed by their greater levels of reduction (Figure 3E). This latter observation supports the concept that destabilization of 5′BS also enhances 5′-end accessibility to ribosomes.

### 3.4. Exploring Alternative Conformations of the 5′UTR of RNA2

Our compensatory mutational analysis clearly established that formation of 5′BS in RNA2 has opposite effects on translation (inhibitory) and replication (stimulatory) (Figure 3). The 5′-end entry of 43S ribosome subunits also implicated 5′-end accessibility as an important feature for efficient translation of RNA2 (Figure 2). When considering translational regulation of RNA2, the requirement for its replication for induction of translation [21] also needs to be integrated into the model. How could RNA2 replication be linked with 5′-end accessibility? One possibility is that some of the newly synthesized RNA2 transcripts adopt an alternative RNA structure with an accessible 5′-end. That is, the 5′UTR, when it first emerges during RNA2 synthesis, could fold into an alternative structure that does not form 5′BS and contains a single-stranded 5′-end. However, because the 5′BS appears to be the dominant (optimal) conformation at equilibrium (Figure 1B), this alternative conformation would be less stable and would eventually refold into the more stable 5′BS conformation.

**Figure 4:**
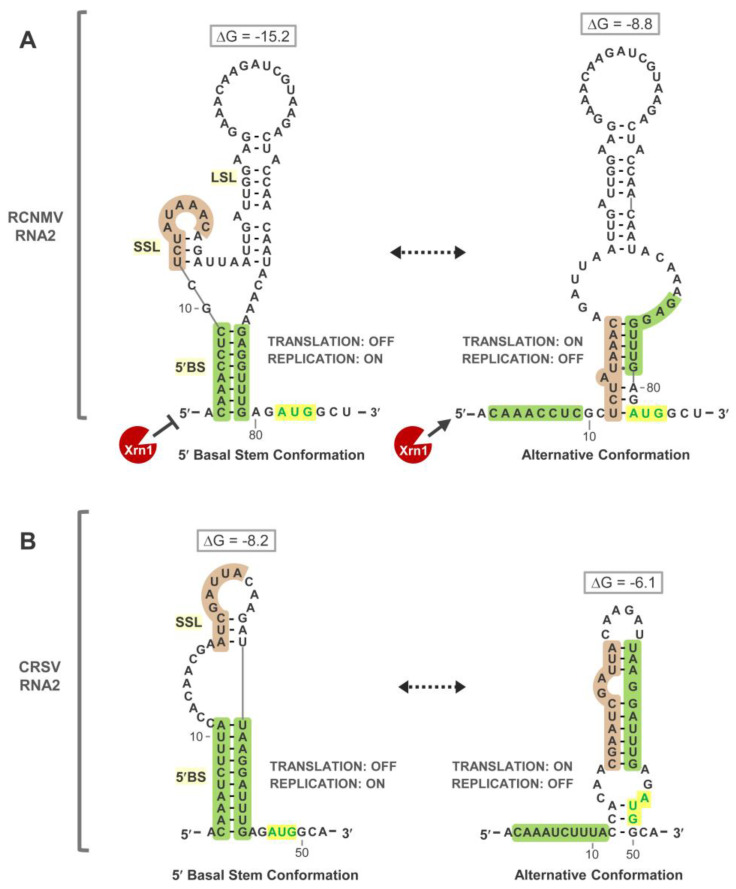
Secondary structural models of candidate 5′UTR conformations in RNA2s of dianthoviruses. (**A**) Candidate 5′UTR conformations for RCNMV RNA2. To the left, the SHAPE-predicted 5′BS conformation that sequesters the 5′-terminal sequence (green shading). To the right, an alternative conformation with different pairing (brown) that results in single-stranded 5′-terminal sequence. Anticipated functional consequences to RNA2 translation and replication are indicted for each conformation, in addition to expected susceptibility to 5′-to-3′ exoribonucleases such as Xrn1. Predicted free energy values (kcal/mol) are shown above each structure. (**B**) Comparable 5′UTR conformations predicted in RNA2 of carnation ringspot virus (CRSV).

To explore this possibility, we examined predicted suboptimal structures for the 5′UTR of RCNMV RNA2. A lower stability alternative conformation was identified in which sequences involved in forming the 3′-half of 5′BS were instead paired to nucleotides located 12 residues from the 5′-terminus, leaving a 5′-terminal segment unpaired (Figure 4A, right panel). Notably, in RNA2 of carnation ringspot virus (CRSV) [39,40,41], a dianthovirus with a shorter and divergent 5′UTR sequence, corresponding structural conformations containing a 5′BS or an unpaired 5′-terminal segment were also predicted for its 5′UTR (Figure 4B). In both cases, the mutually exclusive conformations were predicted to have opposite effects on translation and replication as well as to susceptibility to 5′-to-3′ exoribonucleases such as Xrn1. Based on these congruous observations in closely related viruses, we hypothesized that transient formation of the less stable alternative 5′UTR conformation in nascent RNA2s of RCNMV could allow for efficient loading of 43S ribosome subunits.

### 3.5. Assessing Functionality of RNA2 5′UTR Conformations

**Figure 5:**
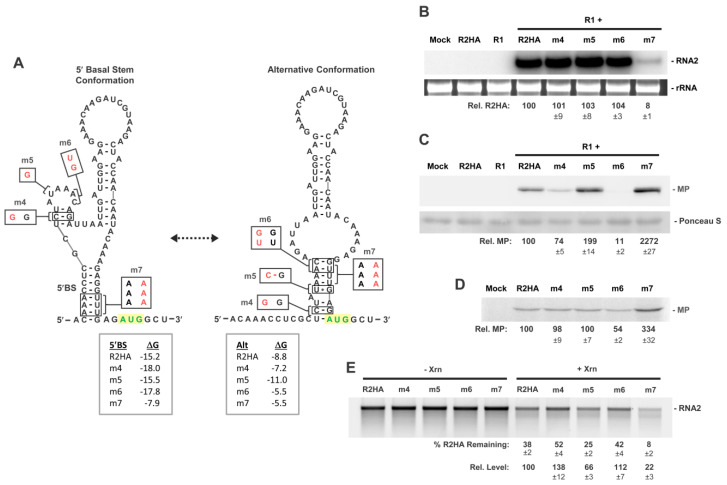
RNA2 5′UTR mutants shifting conformational equilibrium. (**A**) RNA secondary structures for the two proposed conformations in the 5′UTR of RNA2. Substitutions designed to shift the equilibrium of the two conformations are shown in red on both conformations, with predicted free energy values below. (**B**) Northern blot analysis of protoplast infections of wt RNA1 (R1) and RNA2 mutants. (**C**) Corresponding Western blot analysis of protoplast infections depicted in panel (**B**). (**D**) In vitro translation in wheat germ lysate of R2HA and mutants. (**E**) In vitro Xrn1 treatment of R2HA and mutants. Values generated as described in legend of Figure 3.

To evaluate the potential relevance of the alternative conformation (Figure 4A), RCNMV RNA2 mutants were created by introducing nucleotide substitutions in the 5′UTR intended to moderately promote (m5) or inhibit (m4 and m6) the alternative conformation (Figure 5A). Western blot analysis revealed that, relative to R2HA accumulation levels (Figure 5B), a mutation designed to assist stabilization of the alternative conformation in m5 enhanced MP levels (~199%), while two mutants predicted to hinder formation of the alternative conformation in m4 and m6 reduced MP accumulation (~74%, ~11%, respectively) (Figure 5C). Substitutions in m7 obstructed formation of both 5′BS and the alternative structure, and this severely reduced RNA2 levels (~8%) (Figure 5B) while markedly elevating relative MP levels (~2272%) (Figure 5C). This latter observation confirmed our earlier finding on the importance of 5′BS for RNA2 replication (Figure 3) and indicated that formation of the alternative structure is not essential for efficient translation. When tested using in vitro translation assays, the trend observed for m6 and m7, but not m4 and m5, were consistent with in vivo results (Figure 5D), revealing a degree of disparity in the two systems. Results from assessment of the viral transcripts in Xrn1 assays matched the predicted effects of the substitutions on 5′-end accessibility (Figure 5E) and were consistent with corresponding in vivo findings on translation efficiency (Figure 5C), supporting the relevance of a transient alternative structure.

### 3.6. Activity of a Minimal 5′UTR in RNA2

**Figure 6:**
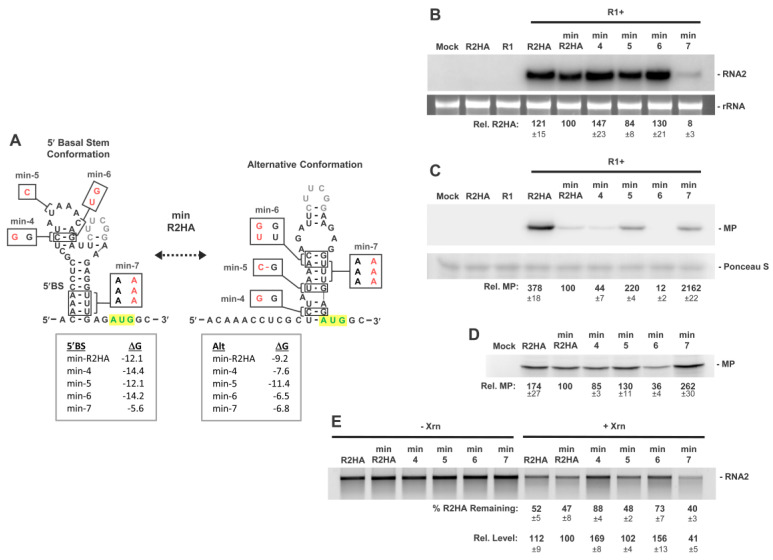
RNA2 minimal 5′UTR mutants shifting conformational equilibrium. (**A**) RNA secondary structures for the two proposed conformations in the minimal 5′UTR of min-R2HA. Substitutions designed to shift the equilibrium of the two conformations are shown in red on both conformations, with predicted free energy values below. (**B**) Northern blot analysis of protoplast infections of wt RNA1 (R1) and min-R2HA mutants. (**C**) Corresponding Western blot analysis of protoplast infections depicted in panel (**B**). (**D**) In vitro translation in wheat germ lysate of min-R2HA and mutants. (**E**) In vitro Xrn1 treatment of min-R2HA and mutants. Values generated as described in legend of Figure 3.

The primary sequences involved in the transition between the 5′BS conformation and the alternative conformation involve the first 20 nts of the 5′UTR and a segment just upstream of the AUG for MP (Figure 4A). The LSL resides between these two segments and could potentially be involved in regulating conversion between the two conformations. To address this possibility, LSL was deleted and replaced by a small linker hairpin, creating min-R2HA, which isolated the core sequences implicated in formation of the two mutually exclusive conformations (Figure 6A). In addition to testing the requirement for LSL, the min-5′UTR limited potential alternate folds that could complicate the interpretation of results. In protoplast infections, min-R2HA accumulated to slightly lower levels than R2HA containing the full-length 5′UTR (Figure 6B) but exhibited about fourfold lower production of MP (Figure 6C). This suggests that removal of LSL shifted the conformational equilibrium towards the 5′BS. Nevertheless, when the same set of modifications that were tested in the full-length 5′UTR (Figure 5A) were introduced into the min-5′UTR (Figure 6A), the corresponding results from protoplast infections in the two mutant sets were consistent. Namely, min-4 and min-6, predicted to impede folding of the alternative structure, reduced relative MP levels (Figure 6C) while increasing RNA2 accumulation (Figure 6B). Conversely, min-5, designed to promote the alternative structure enhanced MP accumulation and decreased RNA2 levels (Figure 6B,C).

Interference with formation of both structures in min-7 yielded low levels of its RNA and high relative levels of MP (Figure 6B,C). Findings from in vitro translation and Xrn1 assays of the min-R2HA transcripts were also in agreement with in vivo results, suggesting that the alternative structure facilitates MP translation (Figure 6D) via increasing 5′-accessibility (Figure 6E), respectively. Collectively, these correlations support a transient conformational change in the 5′UTR that modulates translational efficiency.

### 3.7. Conformational Flexibility in the 5′UTR Is Required for Optimal RNA2 Activities

**Figure 7:**
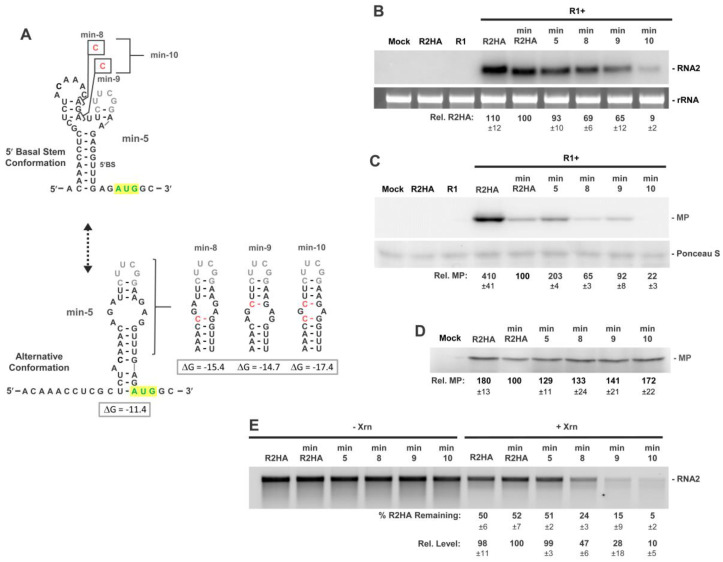
RNA2 minimal 5′UTR mutants assessing stabilization of the alternative conformation. (**A**) RNA secondary structures for the two proposed conformations in min-R2. Substitutions designed to shift the equilibrium toward the alternative conformation are shown in red, with predicted free energy values below. (**B**) Northern blot analysis of protoplast infections of wt RNA1 (R1) and RNA2 mutants. (**C**) Corresponding Western blot analysis of protoplast infections depicted in panel (**B**). (**D**) In vitro translation in wheat germ lysate of min-R2HA and mutants. (**E**) In vitro Xrn1 treatment of min-R2HA and mutants. Values generated as described in legend of Figure 3.

The low level of relative translation from min-R2HA versus R2HA suggested that deleting the LSL likely shifted conformational equilibrium towards the 5′BS (Figure 6C). Conversely, the substitution in min-5, designed to slightly strengthen the alternative structure and shift equilibrium away from 5′BS, led to a twofold increase in relative translation, versus min-R2HA (Figure 6C). We therefore sought to shift equilibrium even further towards the alternative structure by introducing additional alternative structure-stabilizing substitutions into min-5 (Figure 7A), with the expectation that these changes would correspondingly increase 5′-accessibility and relative translation. In protoplast infections, alternative conformation-stabilizing modifications in min-8 through min-10 caused reduced relative levels of MP and RNA2 (Figure 7B,C).

This unexpected result indicated that further stabilization of the alternative structure in infections was doubly detrimental and suggested that a certain balance between the two conformations is required for optimal production of both RNA and protein. Interestingly, when the alternative-structure-stabilized min-R2HAs were tested for translation in vitro, the modifications led to increased MP production (Figure 7D), consistent with their corresponding increased 5′-accessibility in Xrn1 assays (Figure 7E). Accordingly, although the potential for enhanced translation likely existed in these viral RNAs during protoplast infections, the in vivo conditions (e.g., increased sensitivity to 5′-to-3′ exoribonuclease attack) prevented this from occurring.

## 4. Discussion

Here, we have examined further the roles of the 5′BS and 5′UTR in modulating RNA2 replication and translation in RCNMV. Three pieces of evidence support the formation and function of 5′BS: (i) solution structure probing results are consistent with the formation of 5′BS in RCNMV (Figure 1B), (ii) a similar 5′BS is predicted in a divergent dianthovirus species, CRSV (Figure 4), and (iii) compensatory mutational analysis in RCNMV confirmed that base pairing of 5′BS correlates with its function (Figure 3). Previous data revealed that formation of 5′BS facilitates minus-strand RNA2 synthesis in RCNMV [27]. Exactly how 5′BS mediates this initial initiation step in RNA2 replication from the opposite end of the genome is not known, but it could do so by interacting with viral replication proteins p27/88 or host factors that bind to the Y-shaped domain in the 3′UTR of RNA2 [17]. This important role for 5′BS adds to the already notable list of RNA elements involved in promoting RNA2 replication, with the others being a 3′-terminal SL, SL-13 [42,43], the trans-activator (TA) [16,19], and the Y-shaped domain in the 3′UTR [17].

Both genome segments of RCNMV are uncapped, making them more vulnerable to cellular 5′-to-3′ exoribonucleases. In tombusviruses, both secondary and tertiary structural features in the 5′UTR of its uncapped genome form an RNA conformation that protects 5′-terminal nucleotides from Xrn attack by impeding its access [35]. Our results utilizing in vitro Xrn1 assays suggest that 5′BS, by keeping 5′-terminal nucleotides base-paired, performs a similar role in RNA2 end protection (Figure 3). Thus, 5′BS serves a dual function in RNA2 by promoting both its replication and stability. Correspondingly, RNA1 has a localized SL structure at its 5′-terminus that protects it from 5′ attack [23], therefore both genome segments utilize 5′-terminal RNA secondary structures for protection against 5′-to-3′ exoribonucleases.

The balance between RNA2 replication and MP translation must be properly regulated during infections. Our results, along with those of Hyodo et al. [27], indicate that the 5′UTR of RNA2 plays a key role in controlling MP translation and RNA replication. More specifically, our data suggest that a conformational change between two mutually exclusive configurations of the 5′UTR may be involved in regulating the amount and timing of RNA2 and MP accumulation during infections. In hepatitis C virus, an RNA element in the 5′UTR of its plus-strand RNA genome acts as a positive regulator of replication and a negative regulator in translation [44]. A 5′-proximal RNA stem loop was shown to function as an activator of genome replication while concurrently inhibiting translation. We suggest a similar mechanism in RCNMV RNA2 for regulating its replication and translation.

It is proposed that the 5′UTR of RNA2 can assume two mutually exclusive structural configurations: the 5′BS conformation, which prevents translation and permits replication, and the alternative conformation, which promotes translation and inhibits replication (Figure 4A). Transition between these two conformations could allow the 5′UTR to function as a regulatory switch to temporally coordinate the opposing processes of translation and minus-strand synthesis. Evidence supporting the existence of a transient alternative structure include: (i) the prediction of corresponding, but unique, alternative structures in the 5′UTRs of RCNMV and CRSV (Figure 4); (ii) mutations projected to alter the equilibrium of the two structures in RCNMV RNA2 correlate well with predicted effects on translation and replication in vivo and 5′-end accessibility in vitro (Figure 5, Figure 6 and Figure 7); and (iii) a previous study identified nucleotides 11–14 as being critical for promoting translation activity [27], and these residues are involved in formation of the alternative conformation (Figure 4A). The thermodynamic stability of the alternative conformation is estimated to be substantially less than that of the 5′BS conformation (Figure 4A), and this would limit its presence and make it difficult to detect within the 5′UTR structural ensemble (Figure 1B). However, this transient nature is likely important for maintaining integrity of the 5′-terminus of RNA2 during infections, as sustained presence of the alternative structure would make the 5′UTR vulnerable to 5′-to-3′ exoribonucleases, as supported by our data (Figure 7). In addition to differing conformational stabilities, viral or host proteins could also be involved in controlling transitions or stabilization of a particular conformation. The LSL also appears to play some role in facilitating/regulating translational efficiency as MP production was markedly inhibited in its absence (Figure 6).

A previous study showed that replication of RNA2 is required for translation of MP [27], and we suggest that this requirement may be linked to formation of the alternative conformation. As alluded to in the results section, we hypothesize that the 5′UTRs of a proportion of newly synthesized RNA2s initially fold, co-transcriptionally, into the alternative conformation. This would provide a window of opportunity for efficient ribosome loading on the message, before equilibrating back to the more stable 5′BS conformation. It has been shown that translation of RNA2 is preferentially mediated by an isoform of eIF4F, eIFiso4F [26], and it is known that this isoform is less efficient than eIF4F at mediating initiation of translation on messages with significant RNA secondary structure in their 5′UTRs [45]. This latter property could, in part, explain why RNA2 utilizes an alternative conformation, as it would allow for easier access of eIFiso4F and associated translational machinery to the 5′UTR. RNA2′s preference for the isoform 4F would also preclude it from having to directly compete with eIF4F-mediated translation of RNA1 [26].

Based on our results we propose a putative model to describe the involvement of the alternative conformation in regulating RNA2 replication and translation during infections. Upon RCNMV infection, RNA2 released from the virion would harbor the thermodynamically favorable 5′BS structure, which would both protect its 5′UTR from exoribonuclease attack and promote RNA2 replication. The presence of the 5′BS in invading RNA2 genomes would also prevent the ex-virion message from being immediately directed into translation. Unlike for RNA1, which encodes viral replication proteins (p27/88) essential for genome amplification [8], immediate translation of RNA2 is not necessary and in fact could be inhibitory to infections because: (i) production of MP very early in the infection could interfere with genome replication and (ii) immediate translation of RNA2, instead of its replication, could lead to its degradation post-translationally, making it unavailable for replication needed to maintain its presence. Thus, promoting immediate replication of invading RNA2 would help to ensure its reproduction and correspondingly delay translation of MP to a more appropriate later stage of the infection.

Following replication, to allow for translation of MP, newly synthesized RNA2s would form the alternative RNA structure transiently via co-transcriptional folding. This would both promote initiation of a first round of translation by increasing 43S subunit access and inhibit minus-strand synthesis (requiring 5′BS) that would interfere with translation. However, it is unlikely that a single round of translation would be able to provide the levels of MP needed for a successful infection. Thus, after the initial round of translation, additional rounds could follow if the 43S subunit traffic on the 5′UTR acted to delay the formation of 5′BS. Eventually, the nascent RNA2 messages would adopt the more stable 5′BS, and this would allow them to be replicated or packaged into virions. This model provides a possible mechanistic explanation for the observed requirement of RNA2 replication for MP translation during infections and offers new insights into the regulatory strategies utilized during RCNMV infections.
